# A Rare Case of Empagliflozin-Induced Euglycemic Diabetic Ketoacidosis Obscured by Alkalosis

**DOI:** 10.7759/cureus.25818

**Published:** 2022-06-10

**Authors:** Daniel A Fernandez Felix, Gloriana Madrigal Loria, Sapna Sharma, Shorabh Sharma, Carlos E Arias Morales

**Affiliations:** 1 Medicine, St. Barnabas Hospital (SBH) Health System, Bronx, USA; 2 Clinical Medicine, Sophie Davis School of Medicine, City University of New York, Bronx, USA

**Keywords:** diabetic keto-alkalosis, alkalosis, sodium glucose co-transporter 2 inhibitors (sglt2i), euglycemic diabetic ketoacidosis, diabetic ketoacidosis (dka)

## Abstract

Empagliflozin-induced euglycemic diabetic ketoacidosis is a life-threatening metabolic complication of diabetes mellitus characterized by metabolic acidosis, ketonemia, and relatively normal serum glucose levels. We present a rare case of empagliflozin-induced diabetic ketoacidosis obscured by alkalosis. This case report aims to create awareness among clinicians about this entity and consider this diagnosis in their differential, especially in patients taking sodium-glucose co-transporter (SGLT-2) inhibitors who present to the hospital with unspecific symptoms that may not suggest DKA.

## Introduction

Empagliflozin is a sodium-glucose co-transporter inhibitor (SGLT-2i) that has been approved by the Food and Drug Administration (FDA) since August 2014 for the treatment of type II diabetes mellitus (T2DM) [1]. The primary mechanism of action of SGLT-2i is by inhibition of glucose uptake in proximal tubules, leading to glycosuria [2]. Common side effects of this drug class include increased risk of urinary tract infections, genital mycotic infections, and dehydration [3]. In May 2015, regulatory agencies issued a warning that SGLT-2i may cause diabetic ketoacidosis (DKA) with uncharacteristically mild to moderate glucose elevations [3]. DKA-associated with SGLT-2i has rates ranging from 0.16 to 0.76 events per 1,000 patient-years in patients with T2DM [4]. Empagliflozin-induced DKA usually presents with severe metabolic acidosis with elevated anion gap but only mild to moderate hyperglycemia, also known as euglycemic DKA (eDKA). Common precipitants of eDKA include infection, alcohol use, starvation, pregnancy, acute illness, and in patients who are fasting prior to surgeries without maintenance fluids containing dextrose. Appropriate treatment is often delayed as it presents with normal glucose levels in serum; hence, it is of utmost importance that physicians possess a high level of suspicion for eDKA in patients treated with SGLT-2i, especially when the initial presentation is atypical, for example, eDKA presenting with alkalosis instead of acidosis. We describe a case of empagliflozin-induced eDKA in a patient with T2DM who presented with a mixed acid-base disorder with predominant alkalosis.

## Case presentation

A 37-year-old man presented to the emergency department (ED) with a two-day history of nausea and multiple episodes of non-bilious, non-bloody vomiting. The patient had been seen two days prior complaining of similar symptoms and was treated with anti-emetics and was discharged home after symptomatic improvement. The patient denied fever, abdominal pain, and diarrhea. Past medical history was significant for T2DM treated with empagliflozin 25 mg daily for one year. The patient stated compliance with the medication, and the last dose taken was one day before admission. Vital signs on admission reported a heart rate of 97 beats per minute; respiratory rate of 20 breaths per minute, blood pressure of 164/97 mmHg, and a temperature of 98.8^o^F (37.1^o^C); pulse oximetry was 99% on room air. The patient appeared uncomfortable, intermittently sticking fingers down his throat while retching. Oral mucosa was dry, abdomen was soft, non-tender, non-distended. The remainder of the physical exam was unremarkable.

Pertinent biochemical analysis (Table 1) on day one of admission included: a mild increase in glucose, low carbon dioxide, elevated anion gap, serum creatinine at the upper limit of a normal, mild increase in white blood cell count, elevated lactic acid, normal blood osmolality, large ketones, and negative blood alcohol level. Urine analysis showed ketonuria and glucosuria. Initial venous blood gas (VBG) as depicted in Table 2 was remarkable by elevated pH, low partial pressure of carbon dioxide, and low bicarbonate. The patient was admitted to the general medical floor and was started on IV fluids and antiemetics. No antibiotics were prescribed.

**Table 1:** Basic metabolic panel trend Glucose reference range (70-99 mg/dL), carbon dioxide reference range (23-30 mEq/L), sodium reference range (135-145 mEq/L), chloride reference range (96-108 mEq/L), anion gap reference range (7-16 mEq/L)

Date	6/22/2021	6/22/2021	6/23/2021	6/23/2021	6/23/2021	6/23/2021	6/23/2021	6/23/2021	6/23/2021	6/24/2021	6/24/2021	6/24/2021	6/25/2021
Hour	11:16h	15:26h	00:43h	07:50h	12:22h	16:46h	17:00h	21:30h	23:01h	02:00h	06:45h	19:00h	07:37h
Sodium	138	140	141	139	135	137	136	134	137	139	141	137	138
Chloride	95	102	103	104	97	101	100	103	106	107	109	101	101
Carbon dioxide	19	17	19	15	14	14	17	13	15	19	21	23	26
Glucose	141	116	117	125	182	147	147	156	134	147	182	163	171
Anion Gap	24	21	20	24	24	22	19	18	16	13	11	13	11

**Table 2:** Venous blood gas trend PH reference range (7.35-7.45), partial pressure of carbon dioxide reference range (35-45 mm[hg]), bicarbonate reference range (24-30 mEq/L)

Date	6/22/21	6/22/21	6/22/21	6/23/21	6/23/21	6/23/21	6/23/21	6/24/21
Hour	11:16	15:26	22:32	0:43	12:48	16:46	21:57	2:00
PH	7.481	7.342	7.356	7.388	7.349	7.333	7.334	7.362
PCO_2_	28.4	36.4	32.7	32	27.5	30.6	29.5	37.3
Bicarbonate	22.6	19.4	19.2	20.2	16.5	17.2	17.1	21.2

By the second day of the hospital stay, symptoms did not improve with a drop in the pH and bicarbonate (Table 2). Endocrinology and Critical care medicine were consulted, and the patient was transferred to the intensive care unit to initiate IV Insulin infusion as per protocol and IV fluids. Subsequent VBG and basic metabolic panel showed improvement with a gradual increase in serum bicarbonate levels, and the anion gap was closed seven hours after initiation of IV insulin infusion (Tables 1, 2). The patient was transitioned to basal insulin after oral route was tolerated by the patient. Subsequently, laboratory parameters were monitored for 24 hours for DKA relapse. By the fourth day of hospitalization, the patient was discharged home, empagliflozin was discontinued, and the patient was started on Metformin 500 mg daily.

## Discussion

Diabetic ketoacidosis is a life-threatening metabolic complication of DM, traditionally defined by hyperglycemia (>250 mg/dL [>13.9 mmol/L]), ketosis (increased plasma ketones), and anion-gap acidosis [5]. DKA is frequently seen in high-dependency units such as critical care units and the emergency department. eDKA is characterized by severe metabolic acidosis (pH < 7.3), bicarbonate < 18 mEq/L, and anion gap > 12 in the presence of glucose levels < 250 mg/dL with ketonemia [4]. In summary, eDKA can be defined as DKA without marked hyperglycemia.

The pathophysiologic mechanisms behind eDKA include a decrease in insulin secretion or action, a decrease in glucose uptake by the cells, increase in counterregulatory hormones such as glucagon, cortisol, and epinephrine, resulting in increased glucagon to insulin ratio, thereby favoring increased lipolysis, increase in free fatty acids and ketoacidosis. SGLT-2 is a sodium-glucose co-transporter present in the apical surface of proximal renal tubules. SGLT-2 expression is enhanced in T2DM and reabsorbs glucose and sodium. SGLT-2i act by blocking glucose's reabsorption in the proximal convoluted tubule, thereby increasing glucose excretion [6-8]. Ketoacidosis occurs by two central mechanisms, primarily due to the lack of insulin. First, the stimulation of free fatty acid production that travels through the bloodstream to the liver leads to ketogenesis. Furthermore, SGLT-2i stimulates glucagon secretion in pancreatic alpha cells, reducing insulin to glucagon ratio. High glucagon levels interfere with fatty acid metabolism by decreasing the production of malonyl-CoA, eventually promoting beta-oxidation and ketoacid production [9,10].

In our case, the patient presented with a triple acid-base disorder with a final alkalotic pH, obscuring the usual metabolic acidosis seen in eDKA. The metabolic acidosis was caused by ketonemia and lactic acidosis, the concomitant metabolic alkalosis (confirmed by an initial delta anion gap/delta bicarbonate of 2.4) was secondary to persistent vomiting, and the respiratory alkalosis (low pCO_2_ in the setting of high pH) due to hyperventilation. Similar combined acid-base disturbances were described in studies by Kumar et al. and Svart et al. They found patients presenting with intractable vomiting and diabetic keto-alkalosis likely due to volume contraction [11,12]. DKA was not strongly suspected in our patient since serum glucose was 141 mg/dL and alkalotic pH in the VBG. After initial management with antiemetics and IV crystalloids, metabolic alkalosis resolved. However, metabolic acidosis persisted, demonstrated by decreased bicarbonate, elevated anion gap, and serum ketones. This presentation of a triple acid-base disturbance and euglycemia caused a delay in management in our patient. After starting insulin drip, the anion gap closed, and bicarbonate levels normalized, supporting our diagnosis of eDKA. 

Treatment of eDKA is virtually the same as hyperglycemic DKA, consisting of aggressive hydration, insulin, and electrolytes homeostasis. The American Association of Clinical Endocrinologists and American College of Endocrinology recommend minimizing the risk of SGLT-2i associated DKA by avoiding excessive alcohol intake, minimizing starvation or decreased carbohydrate intake, and stopping SGLT-2i at least 24 hours before elective surgery [3,13]. Knowing these triggers will help us adequately prescribe SGLT-2i and withhold them in any situation that might precipitate DKA [14].

## Conclusions

Given the current widespread use of empagliflozin due to its cardioprotective effects in diabetic and non-diabetic patients, the number of cases of eDKA will continue to rise and can be easily missed, especially in patients who are yet not diagnosed with diabetes. Hence, providers must keep eDKA in their differential in patients taking SGLT-2i who present to the outpatient and/or inpatient settings with unspecific symptoms such as nausea and vomiting even if they are seemingly not acidotic. Early recognition of this potential side effect can translate into prompt treatment and shorter hospital length of stay. Patients must be routinely informed of the common and uncommon side effects of empagliflozin (including eDKA) and the potential triggers to decrease the risk of complications.
